# Effect of electroacupuncture versus prucalopride for severe chronic constipation: protocol of a multi-centre, non-inferiority, randomised controlled trial

**DOI:** 10.1186/1472-6882-14-260

**Published:** 2014-07-23

**Authors:** Baoyan Liu, Yang Wang, Jiani Wu, Qian Mo, Weiming Wang, Liyun He, Shiyan Yan, Zhishun Liu

**Affiliations:** Guang’anmen Hospital, China Academy of Chinese Medical Sciences, No.5 Beixiange Street Xicheng District, 100053 Beijing, China; China Academy of Chinese Medical Sciences, Beijing, 100053 China

**Keywords:** Acupuncture, Prucalopride, Severe chronic constipation

## Abstract

**Background:**

Acupuncture is safe and may be effective for severe chronic constipation. The World Gastroenterology Organisation recommends prucalopride for patients for whom previous laxative use failed to provide satisfactory relief.

**Methods/design:**

In this prospective, multi-centre, randomised controlled trial, five hundred sixty patients with severe chronic constipation (two or less spontaneous complete bowel movements per week) from 14 centres will be randomised to receive either electroacupuncture or prucalopride. Participants in the electroacupuncture group will receive electroacupuncture for eight weeks, while participants in the control group will take prucalopride (2 mg once daily) for 32 weeks. The primary outcome measure is the proportion of patients having ≥3 spontaneous, complete bowel movements per week, averaged over week three to eight. The secondary outcome measures include eight items, including the proportion of patients having ≥3 spontaneous, complete bowel movements per week averaged over week 9–32, the proportion of patients with one or more increases in spontaneous, complete bowel movements per week from baseline, mean Bristol Stool Scale, etc. Statistical analysis will include the CMH test, nonparametric tests and t tests.

**Discussion:**

We aimed to compare the effect of electroacupuncture versus prucalopride for severe chronic constipation. The limitation of this study is that participants and acupuncturists will not be blinded.

**Trial registration:**

ClinicalTrials.gov Identifier: NCT02047045.

## Background

Twelve percent of adults have constipation worldwide [1]. In China, the figure is 4%-6% [2, 3]. Severe chronic constipation is defined as two or fewer spontaneous, complete bowel movements (SCBMs) per week, hard stool and a sensation of straining during defecation [4]. Due to their widespread use, laxatives are an available choice for constipated patients. However, a high level of dissatisfaction has been reported for laxatives; specifically, evidence supporting the lasting effect of laxatives is limited [5–7]. A study of PEG3350 showed that 38.4 ± 14.1 days after the cessation of PG3350, 29 of 47 (61.7%) patients needed to take additional constipation treatment interventions [8]. A survey of 744 patients with chronic constipation in Europe showed that almost half were using alternative treatments (homeopathy, massage and acupuncture), and nearly 90% of respondents expressed interests in new therapies [6].

With a history of thousands of years, acupuncture has long been used to treat constipation in China. The systematic review [9] we have published concluded that acupuncture was safe for chronic functional constipation and might improve weekly spontaneous bowel movements (SBMs), the quality of life, and relevant symptoms. However, the evidence was limited by the small sample size and the methodological quality. A pilot study (n = 60) we have finished showed that 31.67% patients had 3 or more weekly SBMs after 8 weeks of treatment. By the third month, 40% patients still had 3 or more weekly SBMs, which reflected the lasting effect (effect post treatment) of acupuncture (to be published).

This report is a protocol of a phase-II trial aiming to evaluate the effect of electroacupuncture (EA) versus prucalopride for severe chronic constipation. The World Gastroenterology Organisation recommends prucalopride for patients for whom previous laxative use failed to provide satisfactory relief [10]. It was also considered effective and safe in a randomised controlled trial conducted in the Asia-Pacific region [11]. Thus, the use of prucalopride as the control drug in this trial is reasonable. The objective is to compare the effect of EA versus prucalopride for severe chronic constipation. We will also observe how many patients will not need additional medicine until the 3^rd^ and 6^th^ month post treatment. The safety and acceptance of EA will be simultaneously evaluated.

## Methods/design

### Study design

**Figure 1:**
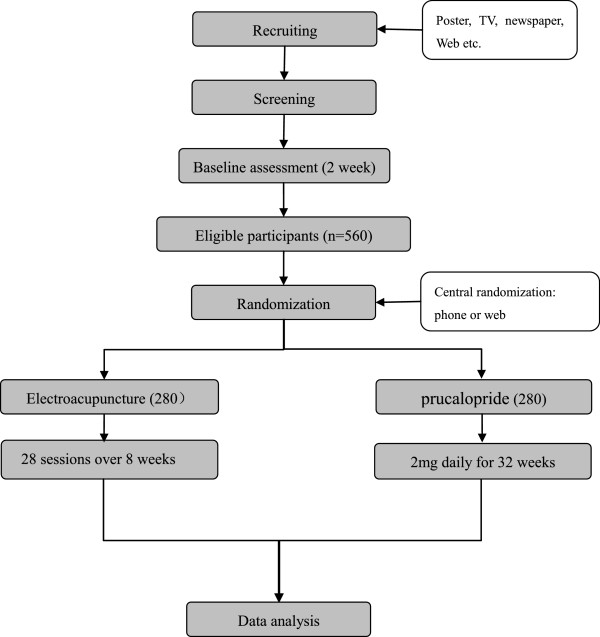
**The flowchart.**

**Figure 2:**
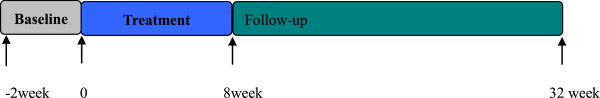
**Time Frame of the Trial.**

This study is a prospective randomised controlled trial. It will be conducted from April 2014 to August 2015 at 14 hospitals around China^a^. Five hundred and sixty participants are planned to be recruited via advertisements in newspapers, television, websites and posters. After being diagnosed by physicians of the gastroenterology or anorectal departments, participants will receive a 2-week long assessment (the stool diary). Randomisation will be performed centrally by the Clinical Evaluation Centre of the China Academy of Chinese Medical Sciences in Beijing, and eligible participants will be randomised into the EA group or the control group. After the baseline assessment has been carried out and the informed consent has been obtained, a randomisation number will be sent to the acupuncturist by phone or online. The statisticians, outcome assessors, and physicians will be blinded to the allocation. The flowchart of the trial is presented in Figure 1. The duration of the trial is 34 weeks, including a 2-week long baseline assessment period (week-2-0), 8-week long treatment period (week 1–8), and 24-week long follow-up period (week 9–32). The time frame of the trial is presented in Figure 2. The trial protocol is in accordance with the principles of the Declaration of Helsinki and has been approved by the review board and ethics committee of the participating hospitals.

### Participants

Five hundred and sixty participants met the Rome III criteria [12] are planned to be recruited.

Inclusion criteria: 1) Primary chronic constipation meeting the Rome III criteria, with ≤2 SCBMs (SCBM refers to the feeling of complete evacuation after a bowel movement without the help of drug or hands in the past 24 hours. Bowel movement beyond 24 hours after the use of medicine/hands also could be considered a SCBM). They should conform to at least one of the following items at the same time: a. a feeling of straining during evacuation (at least once per 4 bowel movements); b. lumpy or hard stools (at least once per 4 bowel movements); c. a feeling of incomplete evacuation after a bowel movement (at least once per 4 bowel movements); d. a feeling of obstruction in the anus/rectum (at least once per 4 bowel movements); e. needing the help of hands for evacuation (at least once per 4 bowel movements). 2) Participants who do not meet the diagnosis of irritable bowel syndrome (IBS). 3) Previous laxative use failed to provide satisfactory relief. 4) Patients aged 18 to 75 years old. 5) Two or fewer SBMs per week during the 2-week long baseline assessment. 6) No use of medicine for constipation during the 2 weeks before enrolment (except rescue medication usage); no acupuncture treatment for constipation in the previous 1 month; no participation in any other on-going trial. 7) Volunteered to join this trial and signed the informed consent.

Exclusion criteria: 1) Irritable bowel syndrome, organic constipation or secondary constipation caused by endocrine, metabolic, nervous, postoperative diseases, or drugs; 2) More than one mushy or watery stool during baseline while not taking laxatives (Bristol stool type 6 or 7); 3) A history of pelvic floor dysfunction; 4) Taking medicine that can influence the intestinal function or induce constipation; 5) Severe haemorrhoid or anal fissure; 6) Patients with severe cardiovascular, hepatic or renal diseases, cognitive impairment, abdominal aortic aneurysm or hepatosplenomegaly, aphasia, mental disorders, or illness that will influence the examination or treatment; 7) Women in the gestation or lactation period; 8) Patients with blood coagulation disorders or taking anticoagulants regularly, such as Warfarin and Heparin; 9) Patients with a cardiac pacemaker carrier.

### Intervention

All participants will be advised of lifestyle modification, such as maintaining their original lifestyle, avoiding fibre-rich food or following a weight-loss diet. Therapies other than the treatment regimen are prohibited during the trial.

#### Treatment group

The acupuncture regimen is based on the Chinese literature from the past 20 years, our pilot trial and specialist consensus. The point regimen (points used for every participants of EA group) includes bilateral ST25, SP14 and ST 37. Moreover, BL33, DU20 and DU24 could be used according to the individual situation. BL 33 could be used for straining and incomplete evacuation. DU20 and DU24 could be used for anxiety and depression.

For ST25 and SP14, the needle will be inserted vertically to the abdominal muscles. The acupuncturist can feel resistance from the tip of the needle when touching the muscles and continues to insert it to a depth of 2–3 mm and stops. For ST 37, the needle will be inserted vertically to a depth of 25 mm. The needle will be manipulated with an even lifting and twisting method three times to achieve the sensation of deqi (patients having a sour and distension sensation). For DU20 and DU24, the needle is obliquely inserted back towards the galea aponeurotica to a depth of 40 mm. The needle is manipulated with an even lifting and twisting method three times to achieve the sensation of deqi. If BL33 is needed, all points could be simultaneously needled when participants are in the lateral decubitus position. The site 0.5-1 cm outside and above the 3rd posterior sacral foramina will serve as the inserting site. The needle is inserted at the inserting site inward and downward at a 30–45 degree angle to a depth of 50–60 mm.

An electric stimulator will be placed on the pair of ST25 and SP14 points with a spare-dense wave, 10/50 Hz, 0.1–1.0 mA (for BL33, the intensity is 0.5-2.0 mA). For ST37, DU20 and DU24, the needles will be manipulated with an even lifting and twisting method every ten minutes, and three manipulations will be used for each participant in all. Patients will be given one session per day for 30 min, 5 sessions/week for the first 2 weeks, and 3 times/week for the next 6 weeks.

#### Control group

Prucalopride Succinate (Janssen S.P.A) will be taken orally at a dose of 2 mg/day before breakfast for 8 weeks. Participants will undergo an electrocardiogram (ECG) at the 8^th^ week, and the drug will be taken for another 24 weeks in the absence of adverse events (like Q-T elongation).

The use of other medicine for constipation will not be allowed during the trial. Bisacodyl (5–10 mg) and Enemia Glycerini (110 ml) can be used for participants who fail to have a bowel movement for 3 or more consecutive days. Furthermore, the use of Bisacodyl and Enemia Glycerini should be recorded on the case report form.

### Outcome measures

The outcome measures are based on the stool diary. The participants complete a stool diary, which is checked by outcome assessors and finally recorded in the Remote Data Capture (RDC) system. The contents of the stool diary include SBMs within 24 hours, straining degree, complete evacuation, stool consistency, use of drugs, etc. Participants will be asked to finish the diaries every day during the baseline assessment period, week 1–8, 11–12, 15–16, 19–20 and 31–32.

This trial includes one primary outcome measure and 8 secondary outcome measures. The primary outcome is the proportion of patients having ≥3 SCBMs/week, averaged over week 3–8. The secondary outcome measures are presented in Table 1. The safety of EA will be evaluated based on various events, including fainting, severe pain, haematoma, local infection, and any feeling of discomfort. The side effects of prucalopride include diarrhoea, nausea, abdominal pain, headache, palpitation, myocardial ischemia (presented in ECG), etc. Any adverse event resulting from EA or adverse drug reaction will be recorded.

**Table 1:** **Secondary outcome measures**

No.	Outcome measure	Time frame	Statistics
1	Proportion of patients having ≥3 SCBMs/week	Week 1–2, week 9–12, week 9–16, week 9–20, week 9-32	CMH test
2	Proportion of patients with one or more increase in SCBMs/week from baseline	Week 1–2, week 3–8, week 9–12, week 9–16, week 9–20, week 9-32	CMH test
3	SCBMs/week and change from baseline	Week 1–2, week 3–8, week 9–12, week 9–16, week 9–20, week 9-32	Nonparametric test
4	SBMs/week and change from baseline in SCBM/week	Week 1–2, week 3–8, week 9–12, week 9–16, week9-20, week 9-32	Nonparametric test
5	Mean Bristol Stool Scale and change from baseline	Baseline, week 1–2, week 3-8	Nonparametric test
6	Straining degree	Week 1–2, week 3–8, week 9–12, week 9–16, week 9–20, week 9-32	Nonparametric test
7	Time to first SCBM	Time from the first treatment to the first SCBM	Nonparametric test
8	Patient assessment of Constipation Quality Of Life (PAC-QOL)	Baseline, week 1–4, week 5-8	*t* test or Mann–Whitney *U* test

### Data collection and quality control

Two researchers will independently input the data using the Remote Data Capture (RDC) software. A Data Verification Plan (DVP) will be established to review the data after input. Two data managers with a medical background will code the medical history, adverse events and drug combinations. After a data review, the data will be submitted to the statistician for final analysis.

A rigorous methodology will be followed to guarantee the quality of the trial. Before commencing the trial, experts in different fields will be invited to review and revise the protocol, and staff from the 14 trial centres will undergo training. A 3-level monitoring system will be established to periodically assess the performance of the trial. The outcome assessments, the completion of case report forms and data management will be closely supervised.

### Sample size and statistical analysis

The sample size will be based on the primary outcome (the proportion of patients having ≥3 SCBMs/week averaged over week 3–8). According to our pilot trial and the literature [4], the proportion of participant having ≥3 SCBMs averaged over week 3–8 was 31.6% for the EA group and 30.9% for the prucalopride group. The figure for the placebo group was 12.0% [4]. Thus, a non-inferiority margin of 10% is reasonable. To assess the non-inferiority between the treatment and control groups, a sample size of 276 for each group will be sufficient, with a one-sided 5% level of significance, a power of 80% and allowing for a 20% dropout. In this trial, we aim to recruit 560 participants, 40 for each hospital.

The data from the 14 centres will be pooled, and the Clinical Evaluation Centre of the China Academy of Chinese Medical Sciences in Beijing will conduct the statistical analysis using the SAS 9.1.3 (SAS Institute, Cary, NC, US) and SPSS Ver.13.0 (SPSS Inc., Chicago, IL, USA) software. The data analysis is based on the intention-to-treat (ITT) population. All statistical analyses will be two-sided tests except for the primary outcome. The level of significance will be established at 0.05. Continuous data will be represented by the mean, standard deviation, median, minimum value, and maximum value; categorical data will be represented by percentages. To compare two independent samples, a t-test or nonparametric test will be used for continuous data, and a chi-square test/the Fisher exact test/nonparametric tests will be used for categorical data. For the primary outcome, the CMH test will be used to avoid the centre effect. The analysis methods for secondary outcomes are presented in Table 1. For safety analysis, the incidence of adverse events will be compared between the two groups using the chi-square test or the Fisher exact test.

## Discussion

Acupuncture has been used to treat constipation in China for thousands of years. Clinical practice and several trials showed that acupuncture is effective for constipation [13, 14]. However, the effect of acupuncture on severe constipation has not been evaluated. A protocol of EA versus sham acupuncture for severe constipation has been published [15].

This is a study design for a non-inferiority, randomised controlled trial aimed to compare the short-term effect (the 3^rd^ to 8^th^ treatment week) of EA with prucalopride for severe chronic constipation. Acupuncture is a non-toxic, economical intervention with minimal adverse effects [16]. If the efficacy of EA is non-inferior to prucalopride, it may be a reasonable option for patients who do not want to experience the side effects of prucalopride. We also want to observe EA’s lasting effect, i.e., EA’s effect at the 3^rd^ and 6^th^ month post treatment. The EA group will be treated for 8 weeks and the prucalopride group will be treated for 32 weeks. A comparison between the two groups after the 8^th^ week could clarify the lasting and dominant effect of EA.

This trial was limited because the participants and acupuncturists could not be blinded. However, the outcome assessors and statistician will be blinded to the allocation.

In conclusion, the trial will compare the effect of EA to that of prucalopride for severe chronic constipation.

### Trial status

Nineteen participants have been recruited.

## Endnote

^a^Guang’anmen Hospital (Beijing), Dongzhimen Hospital(Beijing); West China Hospital (Chengdu), The Third Affiliated Hospital of Zhejiang Chinese Medical University (Hangzhou), Hengyang Hospital of Hunan University of Chinese Medicine (Hengyang), Beijing Hospital of TCM (Beijing), Affiliated Hospita of Shandong University of TCM (Jinan), The First Hospital of Hunan University of Chinese Medicine (Changsha), Hubei Provincial Hospital of TCM(Wuhan), Jiangsu Province Hospital of TCM (Nanjing), Shanxi Province Hospital of TCM (Xi’an), The Hiser Healthcare (Qingdao), Guangdong Provincial Hospital of Chinese Medicine (Guangzhou) and Wuhan Integrated TCM and Western Medicine Hospital (Wuhan).

## Abbreviations

SCBM: Spontaneous, complete bowel movements
SBM: Spontaneous bowel movements
EA: Electroacupuncture
ITT: Intention-to-treat
RDC: Remote data capture.
